# Plasmodesmata without callose and calreticulin in higher plants – open channels for fast symplastic transport?

**DOI:** 10.3389/fpls.2014.00074

**Published:** 2014-03-05

**Authors:** Kirill N. Demchenko, Olga V. Voitsekhovskaja, Katharina Pawlowski

**Affiliations:** ^1^Komarov Botanical Institute, Russian Academy of SciencesSt. Petersburg, Russia; ^2^Department of Ecology, Environment and Plant SciencesStockholm University, Stockholm, Sweden

**Keywords:** cell-to-cell communication, plasmodesmata, desmotubule, calreticulin, callose, callose synthase, pollen

## Abstract

Plasmodesmata (PD) represent membrane-lined channels that link adjacent plant cells across the cell wall. PD of higher plants contain a central tube of endoplasmic reticulum (ER) called desmotubule. Membrane and lumen proteins seem to be able to move through the desmotubule, but most transport processes through PD occur through the cytoplasmic annulus (Brunkard etal., 2013). Calreticulin (CRT), a highly conserved Ca^2+^-binding protein found in all multicellular eukaryotes, predominantly located in the ER, was shown to localize to PD, though not all PD accumulate CRT. In nitrogen-fixing actinorhizal root nodules of the Australian tree *Casuarina glauca*, the primary walls of infected cells containing the microsymbiont become lignified upon infection. TEM analysis of these nodules showed that during the differentiation of infected cells, PD connecting infected cells, and connecting infected and adjacent uninfected cells, were reduced in number as well as diameter (Schubert etal., 2013). In contrast with PD connecting young infected cells, and most PD connecting mature infected and adjacent uninfected cells, PD connecting mature infected cells did not accumulate CRT. Furthermore, as shown here, these PD were not associated with callose, and based on their diameter, they probably had lost their desmotubules. We speculate that either this is a slow path to PD degradation, or that the loss of callose accumulation and presumably also desmotubules leads to the PD becoming open channels and improves metabolite exchange between cells.

## PLASMODESMATA OF HIGHER PLANTS CONTAIN DESMOTUBULES AND ARE USUALLY ASSOCIATED WITH CALRETICULIN

Plasmodesmata (PD) represent membrane-lined channels that link adjacent plant cells across the cell wall and provide symplasmic connectivity, allowing the transfer of metabolites, RNAs, proteins, viruses, and even plastids (Thyssen et al., 2012). PD of higher plants contain a central tube of endoplasmic reticulum (ER) called the central rod or desmotubule. The surfaces of the desmotubule and of the plasma membrane are covered with globular particles interlinked with spokes, thereby stabilizing the internal structure of the PD and also limiting their lumen. Cell-to-cell movement of ER membrane dyes and even – proteins seems to be possible through the desmotubule (Martens et al., 2006; Guenoune-Gelbart et al., 2008), and in spite of its appressed form, molecules of up to 10.4 kDa can move through the ER lumen between neighboring cells in some cases (Barton et al., 2011). However, most transport processes through PD occur through the cytoplasmic annulus, the region between plasma membrane and desmotubule. Early studies suggested a size exclusion limit (SEL) of PD in the order of 1 kDa (Robards and Lucas, 1990), but this can be increased to up to 67 kDa in response to changes in the cytosolic Ca^2+^ concentration or interaction with specific proteins (Oparka et al., 1999; Stadler et al., 2005; Lucas, 2006). PD structure is highly dynamic; e.g., PD morphology can change from simple to branched during the sink source transition in leaves, concomitant with a decrease in SEL (Oparka et al., 1999; Roberts et al., 2001).

PD are assumed to have evolved in multicellular algae several times independently, including in Characeae, the ancestors of higher plants (Raven, 2005). Interestingly, not all multicellular algae have PD (Raven, 2005). The structure of algal PD differs from that of higher plants, most dramatically by the absence of a desmotubule in algal PD (Cook et al., 1997; Cook and Graham, 1999). However, there are reports on desmotubules in PD of Chlorophyceae and Characeae (*Uronema*, *Stigeoclonium*, *Chara*; Marchant, 1976; Brecknock et al., 2011).

Calreticulin (CRT), a highly conserved Ca^2+^-binding protein found in all multicellular eukaryotes examined so far, is predominantly located in the ER (Michalak et al., 1999). CRT was also found in the Golgi (Borisjuk et al., 1998), and in animals also in the cytoplasm of certain cells (Dedhar, 1994), and at the cell surface (Johnson et al., 2001). CRT was shown to localize to PD in maize root apices (Baluška et al., 1999), as well as to PD in suspension cell cultures of tobacco and *Arabidopsis* (Laporte et al., 2003; Bayer et al., 2004), suggesting a role in cell-to-cell transport. This suggestion was supported by the finding that CRT interacts with a viral movement protein (Chen et al., 2005). However, root cap PD do not accumulate CRT (Baluška et al., 1999, 2000). Postmitotic cells of the root epidermis, which like root cap cells are symplasmically isolated, also do not accumulate CRT (Baluška et al., 1999, 2000, 2001), leading to the suggestion that CRT might represent a marker for sink strength. However, CRT is also formed in response to different stresses, and detailed observations led to hypothesis that it represents a universal mediator of fast plasmodesmal closure (Bilska and Sowiński, 2010). It is not quite clear whether CRT is localized in the ER – i.e., near the beginning of the desmotubule – or in the cell wall (Baluška et al., 1999; Bayer et al., 2004); yet, a comparison of the immunolocalization of CRT and callose favors a localization in the ER (Bayer et al., 2004).

## CALLOSE PLAYS A ROLE IN REGULATING THE SEL OF PD

The transport through PD can be regulated by the deposition of callose, a β-1,3-glucan, between the plasma membrane and the wall in the neck region where the cytoplasmic annulus is constricted (Bucher et al., 2001; Simpson et al., 2009). Callose production is catalyzed by callose synthases in the cell wall and is induced by biotic as well as abiotic stresses (Scheible and Pauly, 2004; Benitez-Alfonso and Jackson, 2009). The identification of mutants affected in cell redox homeostasis as well as in intercellular transport, and the observation of changes in symplastic permeability of tissues in response to treatment with oxidants, have been interpreted to suggest that intercellular transport is regulated in response to the production of reactive oxygen species (ROS)* via* callose formation (Benitez-Alfonso and Jackson, 2009; Benitez-Alfonso et al., 2011).

## INFECTED CELLS IN ROOT NODULES OF *C. glauca* REDUCE THEIR PD CONNECTIONS TO ADJACENT CELLS IN THE COURSE OF DEVELOPMENT, WHICH IS ASSOCIATED WITH THE LOSS OF CRT ASSOCIATION

**FIGURE 1:**
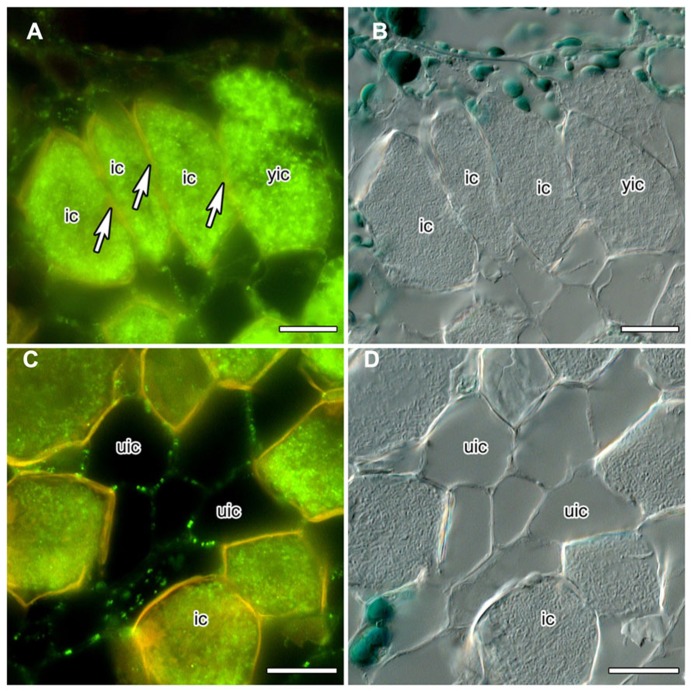
**Immunolocalization of calreticulin (CRT) in longitudinal sections of *C. glauca* nodules embedded in Steedman’s wax (for the method, see Zdyb et al., 2011; for the antibody, see Baluška et al., 1999).** Fluorescence micrographs ** (A,C)** and differential interference contrast micrographs ** (B,D)** are shown. The lignified walls of infected cells fluoresce in yellow under blue light. ** (A,B) ** No CRT labeling is found in walls (see arrow) between infected cells (ic). ** (C,D)** CRT is labeled in walls connecting uninfected cells (uic) in a punctate pattern. Size bars denote 20 μm.

Nitrogen-fixing root nodules, specifically their infected cells that harbor the nitrogen-fixing bacterial microsymbionts which rely on the plant for carbon supply, represent carbon sinks and nitrogen sources. Analysis of the mechanisms of phloem photosynthate partitioning in actinorhizal nodules of the Australian tree *C. glauca* revealed that here, plasmodesmal connections between infected cells, and to a lesser degree between infected and uninfected cells, were reduced during the differentiation of infected cells (Schubert et al., 2013). This concerned the number as well as the diameter of PD. The numbers of PD connecting infected cortical cells were reduced more strongly than the numbers connecting infected to adjacent uninfected cortical cells (by 84 vs. 60%, respectively) but the reduction in diameter was similar in both cases (by 55 vs. 49%, respectively). Furthermore, PD connecting mature infected cortical cells did not accumulate CRT (Schubert et al., 2013; **Figures 1A,B**). CRT labeling was only in rare cases observed for PD connecting infected and adjacent uninfected cells, but was common for PD connecting uninfected cells (Schubert et al., 2013; **Figures 1C,D**).

Under the assumption that CRT is localized in the ER at the opening of the PD, its absence might imply the absence of desmotubules. Desmotubule membranes are the closest juxtaposed lipid bilayers known in nature, 10–15 nm in diameter at their most constricted (Burch-Smith and Zambryski, 2012). Thus, in PD with a diameter of 22 or 26 nm, respectively, the absence of desmotubules should not be surprising, particularly in view of the fact that proteinaceous spokes should protrude from the desmotubule, and globular particles from the plasma membrane in the cytoplasmic sleeve (Burch-Smith and Zambryski, 2012). Loss of desmotubules has also been observed in nematode-parasitized root cortical cells from clover (*Trifolium incarnatum*) and tomato (*Solanum esculentum*), but here this phenomenon was associated with an increase in PD diameter (Hussey et al., 1992). 

## PD OF MATURE INFECTED CELLS OF *C. glauca* NODULES DO NOT SHOW CALLOSE ACCUMULATION

**FIGURE 2:**
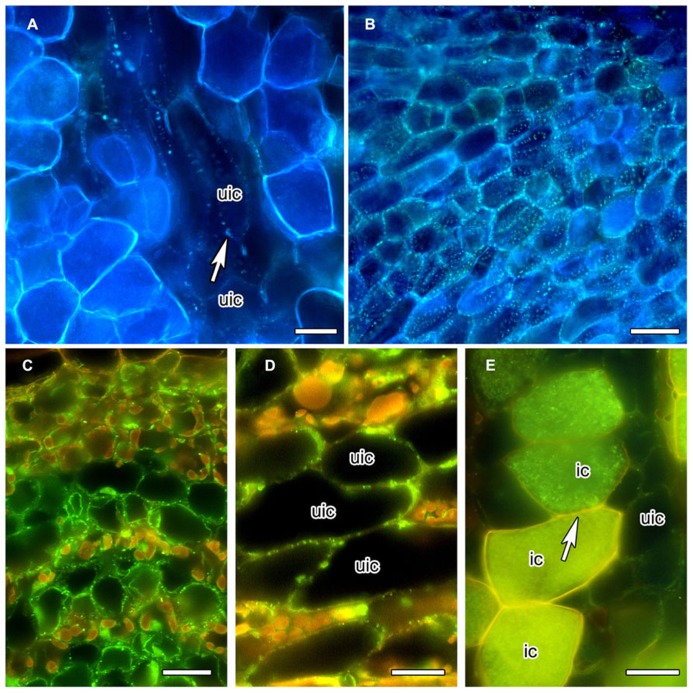
**Anilin Blue staining of callose (A,B) in longitudinal vibratome sections of living *C. glauca* nodules embedded in agarose (Brundrett et al., 1988), and immunolocalization of callose synthase ** (C–E)** in cross sections of fixed nodules embedded in Steedman’s wax (for the method, see Zdyb et al., 2011; for the antibody, see Cai et al., 2011).** Micrographs were taken under fluorescent light. The lignified walls of infected cells show in white under UV light ** (A,B)** and in yellow under blue light ** (C–E)** . ** (A,B)** Punctate Anilin Blue-staining of callose (white fluorescence) is found in the walls connecting uninfected cells (uic; white arrow), but no labeling is found in infected cells [recognizable by their fluorescent cell walls; labeling should be visible because the fluorescence of Anilin Blue, as obvious in ** (B)** which shows a younger area of the cortex before the onset of infection, is more yellowish then the fluorescence of the walls of infected cells]. ** (C)** Callose synthase is detected in the plasma membranes of uninfected cortical cells in the youngest part of the cortex, close to the apical meristem. In the older part of the cortex – ** (D)** shows the outer cortex in the zone of nitrogen fixation – the labeling is less dense. ** (E)** No labeling was seen in the plasma membranes of infected cells (ic; arrow). Size bars denote 20 μm in ** (A–D)** and 25 μm in ** (E)** .

In order to obtain more information on the special features of the PD between infected cells, we analyzed the distribution of callose and of callose synthase. The gradual decrease of PD diameter during the differentiation of infected cortical cells of *C. glauca* nodules was associated with the loss of callose accumulation at PD connecting infected cells, or infected and adjacent uninfected cells, as detected by Aniline blue staining (**Figure 2A**). Aniline blue staining of callose was common for PD connecting uninfected cortical cells (**Figure 2B**). In an attempt to confirm the absence of callose at PD connecting infected cells, an antibody raised against callose synthase from *Nicotiana alata* pollen tubes (Cai et al., 2011) was used. The antibody labeled small granules in the plasma membranes of the youngest cells of the nodule lobe, close to the meristem (**Figure 2C**). Punctate labeling adjacent to the cell walls between uninfected cortical cells in the area of mature infected cells was also found (**Figure 2D**); however, no labeling was observed in walls of infected cells (**Figure 2E**). Since callose synthases are encoded by a gene family (Verma and Hong, 2001), no firm conclusions can be drawn regarding the potential presence of callose at PD connecting infected cells of *C. glauca* nodules; yet, the localization of callose and the immunolocalizations of callose synthase are consistent. 

## MATURE INFECTED CELLS OF *C. glauca* ARE APOPLASTICALLY ISOLATED

Interestingly, *C. glauca* infected cells should depend on symplasmic supply with photosynthates since apoplastic transport pathways a blocked by the impregnation of the walls of infected cells with a very hydrophobic type of lignin (Berg and McDowell, 1988; Schubert et al., 2013). It has been suggested that the properties of the cell wall surrounding PD may restrict the degree to which the microchannels can dilate (Kragler et al., 1998). Therefore, this lignification, which commences upon infection by the microsymbiont, is the most likely explanation for the observed reduction of PD diameter in *C. glauca* infected cells. While many cases are known where lignification or suberization of cell walls does not affect the PD traversing these cell walls, in such cases the PD are organized in primary pit fields, i.e., areas with reduced thickness of the primary wall and without secondary cell wall deposition (Robinson-Beers and Evert, 1991) and with, it seems, a distinctive cell wall composition (Orfila and Knox, 2000), while in infected cells of *C. glauca* nodules, the primary walls are lignified, i.e., the PD traverse the lignified parts of the wall.

## WHAT ARE THE EFFECTS OF THE CHANGES IN PD OF INFECTED CELLS IN *C. glauca* NODULES ON SYMPLASTIC TRANSPORT?

The diameters of PD connecting infected cells with adjacent infected or uninfected cells are significantly reduced compared to those connecting uninfected cells (Schubert et al., 2013). The lack of callose and callose synthase would mean the absence of negative regulation of the SEL of these PD by callose deposition. Similarly, no callose deposition was observed along cell walls between giant cells in nematode feeding sites of tobacco, but callose deposition was found frequently along cell walls toward neighboring cells (Hofmann et al., 2010).

The complete lack of CRT labeling at PD connecting infected cells, and the rarity of CRT labeling at PD connecting infected and adjacent uninfected cells, cannot be linked to the hypothesis that CRT represents a marker for sink strength (Baluška et al., 2001) since these cells, which express sucrose synthase at high levels, are strong sinks (Schubert et al., 2013). Furthermore, the infected cells are microaerobic (Berg and McDowell, 1987; Schubert et al., 2013) and usually, ATP depletion leads to the opening of PD (Cleland et al., 1994). However, the loss of CRT labeling is consistent with the hypothesis that CRT is a mediator of fast plasmodesmal closure. First, the PD closure mechanism involving CRT might not be able to function in PD traversing lignified primary cell walls. Second, since the infected cells are apoplastically isolated, additional symplastic isolation would mean cell death, and therefore should not be too easy to install.

It seems likely the changes in PD traversing the walls of infected cells are linked to the lignin deposition in the primary walls of infected cells. This can be interpreted in two ways. Either, the effect of lignification might be the gradual loss of function of PD, adding symplastic isolation to the apoplastic isolation of infected cells of *C. glauca* nodules. In that case, infection would eventually lead to cell death. Alternatively, the shrinking of the PD diameter could be compensated for by the loss of the desmotubules that might be implied by the loss of CRT labeling. Thanks to the central desmotubule, globular particles on the plasma membrane that lines the channel, and spoke-like connections between the desmotubule and the plasma membrane, the operational diameter of PD is no larger than 2 (Van Bel, 1993) or 3 nm (Kragler, 2013). Thus, the disappearance of the desmotubule could be expected to correlate with the disappearance of the spokes, increasing the operational diameter to one that is higher than of normal PD. Hence, the lack of desmotubules and callose would transform the PD to wide open channels for symplasmic transport. That would be consistent with the only known example for loss of PD desmotubules in plant cells, namely in nematode-parasitized root cortical cells from clover (*T. incarnatum*) and tomato (*S. esculentum*; Hussey et al., 1992).

## PD OF INFECTED CELLS OF *C. glauca* NODULES – ON THE WAY TO COMPLETE CLOSURE, OR OPEN CHANNELS FOR OPTIMIZED SYMPLASTIC TRANSPORT?

In order to test which of these hypotheses is correct, a construct expressing green fluorescent protein (GFP) under control of a promoter specific to uninfected nodule cortical cells could be used. So far, no promoter driving expression specific to uninfected root cortical cells of *C. glauca* nodules has been characterized; however, Perrine-Walker et al. (2011) described an auxin efflux carrier (CgPIN1) that was present specifically in uninfected, not in infected nodule cortical cells. Hence, the promoter of *CgPIN1* could be used to drive the expression of *GFP *in uninfected nodule cortical cells. With a molecular mass of 27 kDa and a Stokes radius of 1.8 nm, GFP can travel through the PD connecting root cortical cells (Stadler et al., 2005) and should also be able to pass through the PD connecting infected with adjacent uninfected cortical cells of *C. glauca.* A β-glucuronidase (GUS) fusion construct with the same promoter could serve as negative control, since with a molecular mass of 68 kDa and a Stokes radius of 3.3 nm (Fisher and Cash-Clark, 2000), GUS cannot travel through most PD (Fukuda et al., 2005). If cytological analysis shows the presence of GFP in infected cells of transgenic nodules, larger GFP constructs could be tested for a precise assessment of the SEL of the PD of infected cells.

## Conflict of Interest Statement

The authors declare that the research was conducted in the absence of any commercial or financial relationships that could be construed as a potential conflict of interest.

## References

[B1] Baluška F. Barlow P. W. Volkmann D. (2000). “Actin and myosin VIII in developing root cells,” in *Actin: A Dynamic Framework for Multiple Plant Cell Functions* eds Staiger C. J. Baluška F. Volkmann D. Barlow P. W. (Dordrecht: Kluwer Academic Publishers) 457–476

[B2] Baluška F. Cvrcková F. Kendrick-Jones J. Volkmann D. (2001). Sink plasmodesmata as gateways for phloem unloading. Myosin VIII and calreticulin as molecular determinants of sink strength? * Plant Physiol.* 126 39–46 10.1104/pp.126.1.39 11351069 PMC1540107

[B3] Baluška F. šamaj J. Napier R. Volkmann D. (1999). Maize calreticulin localizes preferentially to plasmodesmata in root apex. *Plant J.* 19 481–488 10.1046/j.1365-313X.1999.00530.x 10504570

[B4] Barton D. A. Cole L. Collings D. A. Liu D. Y. Smith P. M. Day D. A. (2011). Cell-to-cell transport via the lumen of the endoplasmic reticulum. *Plant J.* 66 806–817 10.1111/j.1365-313X.2011.04545.x 21332847

[B5] Bayer E. Thomas C. L. Maule A. J. (2004). Plasmodesmata in *Arabidopsis thaliana* suspension cells. *Protoplasma* 223 93–102 10.1007/s00709-004-0044-8 15221514

[B6] Benitez-Alfonso Y. Jackson D. (2009). Redox homeostasis regulates plasmodesmal communication in *Arabidopsis* meristems. *Plant Signal. Behav.* 4 655–659 10.4161/psb.4.7.8992 19820302 PMC2710567

[B7] Benitez-Alfonso Y. Jackson D. Maule A. (2011). Redox regulation of intercellular transport. *Protoplasma* 248 131–140 10.1007/s00709-010-0243-4 21107619

[B8] Berg R. H. McDowell L. (1987). Endophyte differentiation in *Casuarina *actinorhizae. *Protoplasma* 136 104–117 10.1007/BF01276359

[B9] Berg R. H. McDowell L. (1988). Cytochemistry of the wall of infected cells in *Casuarina* actinorhizae. *Can. J. Bot.* 66 2038–2047

[B10] Bilska A. Sowiński P. (2010). Closure of plasmodesmata in maize (*Zea mays*) at low temperature: a new mechanism for inhibition of photosynthesis. *Ann. Bot.* 106 675–686 10.1093/aob/mcq169 20880933 PMC2958785

[B11] Borisjuk N. Sitailo L. Adler K. Malysheva L. Tewes A. Borisjuk L. (1998). Calreticulin expression in plant cells: developmental regulation, tissue specificity and intracellular distribution. *Planta* 206 504–514 10.1007/s004250050427 9821685

[B12] Brecknock S. Dibbayawan T. P. Vesk M. Vesk P. A. Faulkner C. Barton D. A. (2011). High resolution scanning electron microscopy of plasmodesmata. *Planta* 234 749–758 10.1007/s00425-011-1440-x 21626150

[B13] Brundrett M. C. Enstone D. E. Peterson C. A. (1988). A berberine-aniline blue fluorescent staining procedure for suberin, lignin, and callose in plant tissue. *Protoplasma* 146 133–142 10.1007/BF01405922

[B14] Brunkard J. O. Runkel A. M. Zambryski P. C. (2013). Plasmodesmata dynamics are coordinated by intracellular signaling pathways. *Curr. Opin. Plant Biol.* 16 614–620 10.1016/j.pbi.2013.07.007 23978390 PMC3828052

[B15] Bucher G. L. Tarina C. Heinlein M. Di Serio F. Meins F. Jr. Iglesias V. A. (2001). Local expression of enzymatically active class I β-1,3-glucanase enhances symptoms of TMV infection in tobacco. *Plant J.* 28 361–369 10.1046/j.1365-313X.2001.01181.x 11722778

[B16] Burch-Smith T. M. Zambryski P. C. (2012). Plasmodesmata paradigm shift: regulation from without versus within. *Annu. Rev. Plant Biol.* 63 239–260 10.1146/annurev-arplant-042811-105453 22136566

[B17] Cai G. Faleri C. Del Casino C. Emons A. M. Cresti M. (2011). Distribution of callose synthase, cellulose synthase, and sucrose synthase in tobacco pollen tube is controlled in dissimilar ways by actin filaments and microtubules. *Plant Physiol.* 155 1169–1190 10.1104/pp.110.171371 21205616 PMC3046577

[B18] Chen M. H. Tian G. W. Gafni Y. Citovsky V. (2005). Effects of calreticulin on viral cell-to-cell movement. *Plant Physiol.* 138 1866–1876 10.1104/pp.105.064386 16006596 PMC1183378

[B19] Cleland R. E. Fujiwara T. Lucas W. J. (1994). Plasmodesmal mediated cell-to-cell transport in wheat roots is modulated by anaerobic stress. *Protoplasma* 178 81–85 10.1007/BF01404123 11540962

[B20] Cook M. E. Graham L. E. (1999). “Evolution of plasmodesmata,” in *Plasmodesmata: Nanochannels with Megatasks* eds van Bel A. Kesteren C. (Berlin: Springer Verlag) 101–117

[B21] Cook M. Graham L. Botha C. Lavin C. (1997). Comparative ultrastructure of plasmodesmata of *Chara* and selected bryophytes: toward an elucidation of the evolutionary origin of plant plasmodesmata. *Am. J. Bot.* 84 1169–1178 10.2307/2446040 21708671

[B22] Dedhar S. (1994). Novel functions for calreticulin: interaction with integrins and modulation of gene expression? *Trends Biochem. Sci.* 19 269–271 10.1016/0968-0004(94)90001-9 8048166

[B23] Fisher D. B. Cash-Clark C. E. (2000). Sieve tube unloading and post-phloem transport of fluorescent tracers and proteins injected into sieve tubes via severed aphid stylets. *Plant Physiol.* 123 125–137 10.1104/pp.123.1.125 10806231 PMC58988

[B24] Fukuda A. Fujimaki S. Mori T. Suzui N. Ishiyama K. Hayakawa T. (2005). Differential distribution of proteins expressed in companion cells in the sieve element-companion cell complex of rice plants. *Plant Cell Physiol.* 46 1779–1786 10.1093/pcp/pci190 16120685

[B25] Guenoune-Gelbart D. Elbaum M. Sagi G. Levy A. Epel B. L. (2008). Tobacco mosaic virus (TMV) replicase and movement protein function synergistically in facilitating TMV spread by lateral diffusion in the plasmodesmal desmotubule of *Nicotiana benthamiana*. *Mol. Plant Microbe Interact.* 21 335–345 10.1094/MPMI-21-3-0335 18257683

[B26] Hofmann J. Youssef-Banora M. de Almeida-Engler J. Grundler F. M. (2010). The role of callose deposition along plasmodesmata in nematode feeding sites. *Mol. Plant Microbe Interact.* 23 549–557 10.1094/MPMI-23-5-0549 20367463

[B27] Hussey R. S. Mims C. W Westcott S. W. III. (1992). Ultrastructure of root cortical cells parasitized by the ring nematode *Criconemella xenoplax*. *Protoplasma* 167 55–65 10.1007/BF01353581

[B28] Johnson S. Michalak M. Opas M. Eggleton P. (2001). The ins and outs of calreticulin: from the ER lumen to the extracellular space. *Trends Cell Biol.* 11 122–129 10.1016/S0962-8924(01)01926-2 11306273

[B29] Kragler F. (2013). Plasmodesmata: intercellular tunnels facilitating transport of macromolecules in plants. *Cell Tissue Res.* 352 49–58 10.1007/s00441-012-1550-1 23370600

[B30] Kragler F. Monzer J. Shash K. Xoconostle-Cázares B. Lucas W. J. (1998). Cell-to-cell transport of proteins: requirement for unfolding and characterization of binding to a putative plasmodesmal receptor. *Plant J.* 15 367–381 10.1046/j.1365-313X.1998.00219.x

[B31] Laporte C. Vetter G. Loudes A. M. Robinson D. G. Hillmer S. Stussi-Garaud C. (2003). Involvement of the secretory pathway and the cytoskeleton in intracellular targeting and tubule assembly of Grapevine fanleaf virus movement protein in tobacco BY-2 cells. *Plant Cell* 15 2058–2075 10.1105/tpc.013896 12953111 PMC181331

[B32] Lucas W. J. (2006). Plant viral movement proteins: agents for cell-to-cell trafficking of viral genomes. *Virology* 344 169–184 10.1016/j.virol.2005.09.026 16364748

[B33] Marchant H. J. (1976). “Plasmodesmata in algae and fungi,” in *Communication in Plants: Studies in Plasmodesmata* eds Gunning B. E. S. Robards A. W. (Berlin: Springer Verlag) 59–80 10.1007/978-3-642-66294-2_3

[B34] Martens H. J. Roberts A. G. Oparka K. J. Schulz A. (2006). Quantification of plasmodesmatal endoplasmic reticulum coupling between sieve elements and companion cells using fluorescence redistribution after photobleaching. *Plant Physiol.* 142 471–480 10.1104/pp.106.085803 16905664 PMC1586037

[B35] Michalak M. Corbett E. F. Mesaeli N. Nakamura K. Opas M. (1999). Calreticulin: one protein, one gene, many functions. *Biochem. J.* 344 281–292 10.1042/0264-6021:3440281 10567207 PMC1220642

[B36] Oparka K. J. Roberts A. G. Boevink P. Santa Cruz S. Roberts I. Pradel K. S. (1999). Simple, but not branched, plasmodesmata allow the nonspecific trafficking of proteins in developing tobacco leaves. *Cell* 97 743–754 10.1016/S0092-8674(00)80786-2 10380926

[B37] Orfila C. Knox J. P. (2000). Spatial regulation of pectic polysaccharides in relation to pit fields in cell walls of tomato fruit pericarp. *Plant Physiol.* 122 775–781 10.1104/pp.122.3.775 10712541 PMC58913

[B38] Perrine-Walker F. Doumas P. Lucas M. Vaissayre V. Beauchemin N. J. Band L. R. (2011). Auxin carriers localization drives auxin accumulation in plant cells infected by Frankia in *Casuarina glauca* actinorhizal nodules. *Plant Physiol.* 154 1372–1380 10.1104/pp.110.163394 20826704 PMC2971613

[B39] Raven J. A. (2005). “Evolution of plasmodesmata,” in *Annual Plant Reviews, Plasmodesmata* Vol. 18 ed. Oparka K. J. (Hoboken:Wiley-Blackwell) 33–53

[B40] Robards A. W. Lucas W. J. (1990). Plasmodesmata. *Annu. Rev. Plant Physiol. Plant Mol. Biol.* 41 369–419 10.1146/annurev.pp.41.060190.002101

[B41] Roberts I. M. Boevink P. Roberts A. G. Sauer N. Reichel C. Oparka K. J. (2001). Dynamic changes in the frequency and architecture of plasmodesmata during the sink-source transition in tobacco leaves. *Protoplasma* 218 31–44 10.1007/BF01288358 11732318

[B42] Robinson-Beers K. Evert R. F. (1991). Ultrastructure of and plasmodesmatal frequency in mature leaves of sugarcane. *Planta* 184 291–306 24194146 10.1007/BF00195330

[B43] Scheible W.-R. Pauly M. (2004). Glycosyltransferases and cell wall biosynthesis: novel players and insights. *Curr. Opin. Plant Biol.* 7 285–295 10.1016/j.pbi.2004.03.006 15134749

[B44] Schubert M. Koteyeva N. K. Zdyb A. Santos P. Voitsekhovskaja O. V. Demchenko K. N. (2013). Lignification of cell walls of infected cells in *Casuarina glauca* nodules is accompanied by degradation of plasmodesmata, but infected cells depend on symplastic sugar supply. *Physiol. Plant.* 147 524–540 10.1111/j.1399-3054.2012.01685.x 22924772

[B45] Simpson C. Thomas C. Findlay K. Bayer E. Maule A. J. (2009). An *Arabidopsis* GPI-anchor plasmodesmal neck protein with callose binding activity and potential to regulate cell-to-cell trafficking. *Plant Cell* 21 581–594 10.1105/tpc.108.060145 19223515 PMC2660613

[B46] Stadler R. Wright K. M. Lauterbach C. Amon G. Gahrtz M. Feuerstein A. (2005). Expression of GFP-fusions in *Arabidopsis* companion cells reveals non-specific protein trafficking into sieve elements and identifies a novel post-phloem domain in roots. *Plant J.* 41 319–331 10.1111/j.1365-313X.2004.02298.x 15634207

[B47] Thyssen G. Svab Z. Maliga P. (2012). Cell-to-cell movement of plastids in plants. *Proc. Natl. Acad. Sci. U.S.A.* 109 2439–2443 10.1073/pnas.1114297109 22308369 PMC3289365

[B48] Van Bel A. J. E. (1993). The transport phloem. Specifics of its functioning. * Prog. Bot.* 54 134–150

[B49] Verma D. P. Hong Z. (2001). Plant callose synthase complexes. *Plant Mol. Biol.* 47 693–701 10.1023/A:1013679111111 11785931

[B50] Zdyb A. Demchenko K. N. Heumann J. Mrosk C. Grzeganek P. Göbel C. (2011). Jasmonate biosynthesis in legume and actinorhizal nodules. *New Phytol.* 189 568–579 10.1111/j.1469-8137.2010.03504.x 20964693

